# Emergence of *Acholeplasma laidlawii* Infection in Ducks: Isolation and Colonization Potential

**DOI:** 10.3390/vetsci13101056

**Published:** 2026-10-08

**Authors:** Junfeng Lv, Qiaoya Zhao, Yanhan Liu, Min Kang, Bai Wei, Yufeng Li, Huaiying Xu

**Affiliations:** 1 Poultry Institute, Shandong Academy of Agricultural Sciences, Jinan 250100, China; nkyljf@163.com (J.L.); zqya1990@163.com (Q.Z.); liyufeng@saas.ac.cn (Y.L.); 2 Shandong Provincial Key Laboratory of Livestock and Poultry Breeding, Jinan 250100, China; 3 Shandong Provincial Center for Animal Disease Control, Jinan 250100, China; vetlyh@163.com; 4 Department of Infectious Diseases and Avian Diseases, College of Veterinary Medicine and Research Division of Influenza Korea Zoonosis Research Institute, Chonbuk National University, Jeonju 54989, Republic of Korea; minkang@jbnu.ac.kr (M.K.); weibai.vet@jbnu.ac.kr (B.W.)

**Keywords:** ducks, air sac lesions, *Acholeplasma laidlawii*, low pathogenicity, colonization

## Abstract

*Acholeplasma laidlawii* is a *Mycoplasma* species widely distributed in the environment. It is often regarded as an opportunistic pathogen capable of causing respiratory infections in animals and humans. To date, only a few studies have reported *A. laidlawii* infection in poultry. In the present study, an *A. laidlawii* strain was isolated from the lungs and trachea of diseased ducks presenting with air sac lesions. Antimicrobial susceptibility testing revealed that the isolate was susceptible to tylvalosin, tylosin, and valnemulin, with relatively low minimum inhibitory concentrations (MICs). The pathogenicity of the isolate was evaluated using specific-pathogen-free (SPF) ducks via subcutaneous inoculation. No clinical signs were observed during the experimental period. Oral and cloacal swabs, as well as tissue samples, were tested by PCR. The results showed that the pathogen was detectable in swabs from 5 to 21 days post-inoculation (dpi), and all lung and tracheal samples from infected ducks tested positive. Histopathological examination of the lungs and trachea revealed pathological changes in both tissues. Collectively, these findings demonstrated that *A. laidlawii* was capable of colonizing ducks and inducing mild histopathological changes, suggesting that this pathogen might pose an epidemiological risk in duck farming. Furthermore, these results indicate that *A. laidlawii* could colonize ducks, underscoring the need for continued surveillance to monitor for emerging pathogens in ducks.

## Simple Summary

With the rapid expansion of the duck industry in China, disease prevention and control has become a major constraint on the industry’s development. In recent years, numerous pathogens have exhibited cross-species transmission, including *Tembusu* virus and *Riemerella anatipestifer*, highlighting the need for heightened surveillance of pathogen spread. *Acholeplasma laidlawii*, a species of *mycoplasma*, is widely distributed in the environment and is generally considered an opportunistic animal pathogen. Nevertheless, its infection in ducks has not been documented to date. In this study, we report, for the first time, the isolation of an *A. laidlawii* strain from diseased ducks. Experimental infection in specific-pathogen-free (SPF) ducks confirmed that *A. laidlawii* can establish colonization in ducks. These findings suggested that *A. laidlawii* was capable of colonizing ducks, underscoring the need to establish appropriate surveillance and control measures to monitor the emergence of associated diseases.

## 1. Introduction

*Acholeplasma laidlawii* is a ubiquitous *Mycoplasma* belonging to the class *Mollicutes*, order *Acholeplasmatales*, and family *Acholeplasmataceae*. Like other members of *Mollicutes*, *A. laidlawii* is a cell-wall-deficient bacterium with a reduced genome that has evolved toward a parasitic lifestyle, exhibiting a high dependency on the host for essential metabolites [1,2]. The genome of *A. laidlawii* consists of a single circular chromosome of approximately 1.5 Mbp, with a low GC content of 31% [3]. This bacterium is widely distributed in the environment, including soil, compost, and wastewater, as well as in the tissues of humans, animals, and plants [1]. In humans and animals, *A. laidlawii* acts as a commensal or opportunistic pathogen, causing diseases such as mastitis, arthritis, keratoconjunctivitis, and respiratory infections [4,5,6]. Owing to its small size and lack of a cell wall, the control of *A. laidlawii* infections presents a significant challenge.

Since their emergence in the early 1950s, mycoplasmas have been recognized as the primary etiological agents of chronic respiratory disease in poultry worldwide [7,8,9,10]. Subsequently, numerous mycoplasma strains have been identified in various avian species, including chickens, turkeys, pheasants, sparrows, pigeons, ducks, and geese, thereby exacerbating the associated economic burden [11,12,13,14,15,16]. *A. laidlawii* was first isolated from wastewater in 1936 and was among the first mycoplasma to be successfully cultured on an artificial growth medium [17,18]. Earlier studies demonstrated that *A. laidlawii* strains are pathogenic in goslings, causing fibrinous airsacculitis, peritonitis, and perihepatitis. Moreover, cell-based assays revealed that *A. laidlawii* induces marked pyknosis in chicken embryo fibroblasts [19]. Despite these findings, few clinical cases of *A. laidlawii* infection have been reported in poultry, particularly in ducks.

In the present study, diseased ducks presenting with air sac lesions were diagnosed, and *A. laidlawii* strains were isolated from their lungs and trachea. The pathogenicity of these isolates was subsequently evaluated using specific-pathogen-free (SPF) ducks. Collectively, our findings suggested that *A. laidlawii* could colonize ducks, and continued surveillance is warranted to monitor the appearance of new pathogens in ducks.

## 2. Materials and Methods

### 2.1. Sample Collection

**Figure 1:**
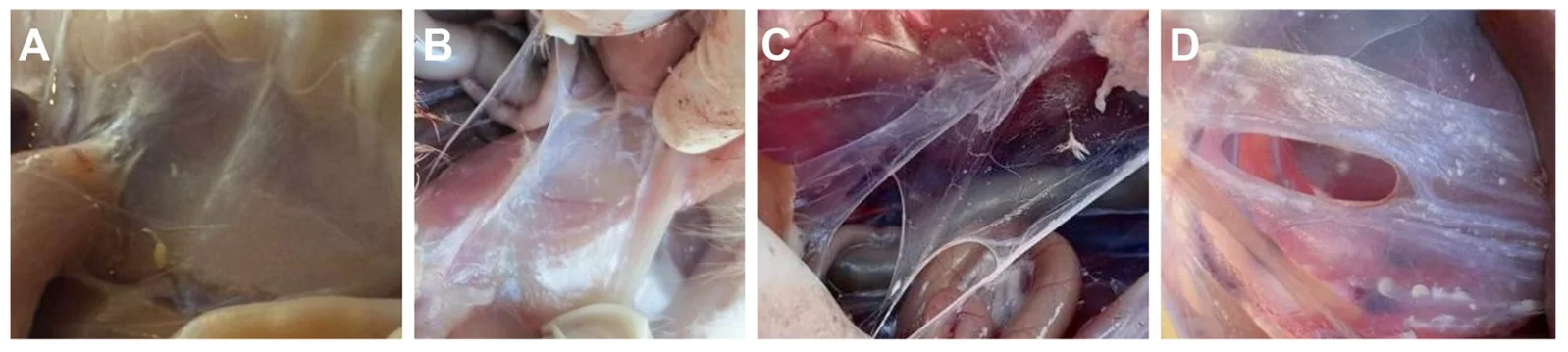
Air sac lesions in ducks with different ages. (**A**) 5-day-old ducks. (**B**) 11-day-old ducks. (**C**) 23-day-old ducks. (**D**) 25-day-old ducks.

Tissue samples (livers, lungs, air sacs, tracheas) were collected from duck farms in Heibei Province, where ducks exhibited respiratory symptoms (Table S1). Postmortem examination of diseased ducks revealed a spectrum of air sac lesions of varying severity. The affected air sacs exhibited sporadic white caseous material, frothy turbidity, and increasingly abundant white caseous deposits. In more severe cases, there was continued accumulation of turbidity and caseous material, along with the sporadic appearance of diffusely distributed yellow caseous exudate. Yellow caseous exudate was present to varying degrees, and in the most severely affected birds, pronounced respiratory signs were observed (Figure 1).

### 2.2. Detection of Common Pathogens

Total DNA/RNA were extracted from homogenized tissues using the VAMNE Magnetic Pathogen DNA/RNA Kit (Vazyme, Nanjing, China), and the DNA/RNA concentration and purity were assessed spectrophotometrically using a NanoDrop One system (Thermo Fisher, Shanghai, China). DNA pathogens were detected using 2 × Rapid Taq Master Mix (Vazyme, Nanjing, China), whereas RNA pathogens were detected using an AccurSTART One Step RT-PCR Kit (Vazyme, Nanjing, China). Both assays were conducted according to the manufacturer’s instructions, with an annealing temperature of 55 °C. Primers specific to avian influenza virus (AIV), avian metapneumovirus (aMPV), Duck Adenovirus (DAdV), Duck Plague Virus (DPV), Infectious Bronchitis Virus (IBV), Duck Circovirus (DuCV), Mollicutes, and *Riemerella anatipestifer* (RA) were designed and are listed in Table 1. PCR products were analyzed using gel electrophoresis.

### 2.3. Isolation of Mycoplasma Strains

Air sacs were collected and homogenized in modified Frey’s liquid medium (pH 7.8, with penicillin (2000 IU/mL), 10% horse serum, 0.01% NAD (coenzyme I), 0.01% L-cysteine, and 0.04% arginine). The homogenate was then incubated at 37 °C until the color of the culture medium changed from red to orange or yellow. The mycoplasma strains were purified on Frey’s solid medium at 37 °C under 5% CO_2_ for four days. The morphology of the strains was subsequently observed. The strains were identified by PCR using primers targeting the mycoplasma 16S rRNA gene, and the PCR products were sequenced by Sangon Biotech Co., Ltd. (Qingdao, China). Sequence homology searches were performed using the NCBI BLAST (v2.2.28) database. The strains were cultured, and their titers were measured and are expressed as CCU/mL as previously described [20].

### 2.4. Phylogenetic Analysis

To determine the genetic relationships of the new isolated *A. laidlawii* strain with the known sequences of other *A. laidlawii* strains reported previously, the identities of the nucleotide sequence of 16S rRNA were aligned using the ClustalW matrix (MegAlign) with Lasergene 7.0 software. Phylogenetic trees were constructed via the maximum likelihood method implemented in the MEGA version 6.0 program by taking 1000 bootstrap replications.

### 2.5. Antimicrobial Susceptibility Testing

The antimicrobial susceptibility of the isolate was determined by the minimum inhibitory concentration (MIC) method using a panel of commonly used antimicrobial agents. The MIC assay was performed in 96-well microtiter plates following previously established protocols [20]. The mycoplasma culture was diluted in mycoplasma broth medium to achieve a final inoculum concentration in the range of 10^3^ to 10^5^ CCU/mL. Two-fold serial dilutions of each antimicrobial agent were prepared in 100 μL of mycoplasma broth medium (pH 7.8). Following the completion of antibiotic dilution, 100 μL of the diluted mycoplasma culture was inoculated into each well. Culture medium without antibiotics and antibiotics without inoculum were included as controls. The plates were incubated at 37 °C and examined daily. The MIC was defined as the lowest concentration of antimicrobial that prevented a visible color change of the phenol red indicator, and was recorded when the color of the negative control (growth control) had just turned from red to orange-yellow.

### 2.6. Animal Experiments

SPF ducks were purchased from Shandong Haotai Laboratory Animal Breeding Co., Ltd. (Jinan, China). Twenty 1-day-old SPF ducks were allocated into two groups (10 ducks per group) and housed in isolators under controlled temperature conditions, with ad libitum access to feed and water. Each duck in the infected group was subcutaneously inoculated with 0.5 mL of *A. laidlawii* culture at a dose of 10^9^ CCU. Ducks in the control group received 0.5 mL of sterile phosphate-buffered saline (PBS) via the same route. All ducks were monitored for 21 days, and morbidity and mortality were recorded and calculated. Cloacal and oral swabs were collected at 1, 3, 5, 7, 9, 11, 14, and 21 days post-inoculation (dpi) and tested by PCR using *A. laidlawii*-specific primers. At the end of the experiment, all ducks were euthanized by immediate exsanguination following a single manual blow with a blunt instrument to the frontoparietal region, and subjected to postmortem examination to evaluate air sac lesions. Tracheas and lungs were collected for histopathological examination, which were conducted by Servicebio Co., Ltd. (Wuhan, China). Tissue samples from the trachea, lungs, liver, spleen, heart, and brain were collected for PCR-based detection of bacteria, following the procedures described above.

## 3. Results

### 3.1. A. laidlawii Was Detected from Tissues of Diseased Ducks

**Figure 2:**
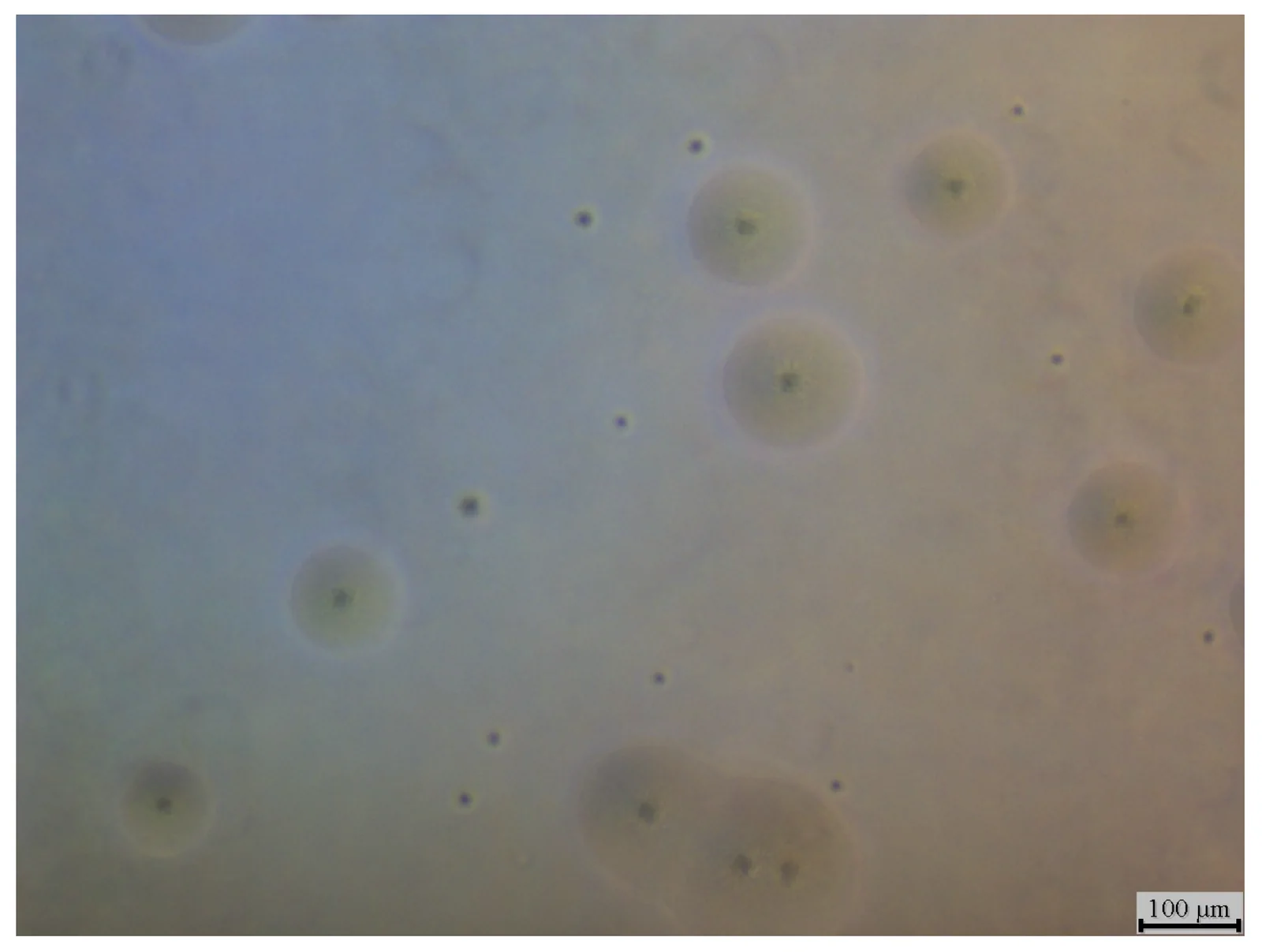
Morphology of isolated colonies on Frey’s solid medium under a low-power microscope (40× magnification). Colonies exhibited “fried-egg” characteristics, with a dense, raised center, and were 0.1–0.2 mm in diameter.

PCR analysis revealed that samples tested negative for AIV, aMPV, DAdV, DPV, IBV, DuCV, and RA, whereas a positive result was obtained with primers targeting the class *Mollicutes*. Following the mycoplasma isolation procedure, colonies exhibiting a small, circular, smooth, and transparent morphology appeared. Under a low-power microscope (40× magnification), typical “fried-egg” colonies characteristic of mycoplasma—with a dense, raised center—were observed, measuring 0.1–0.2 mm in diameter (Figure 2). Based on 16S rRNA gene sequencing of the colonies, the isolate was identified as *A. laidlawii*. The strain was subsequently purified and designated as Duck.HeBei.M9.2025.

### 3.2. Phylogenetic Relationship Analysis

**Figure 3:**
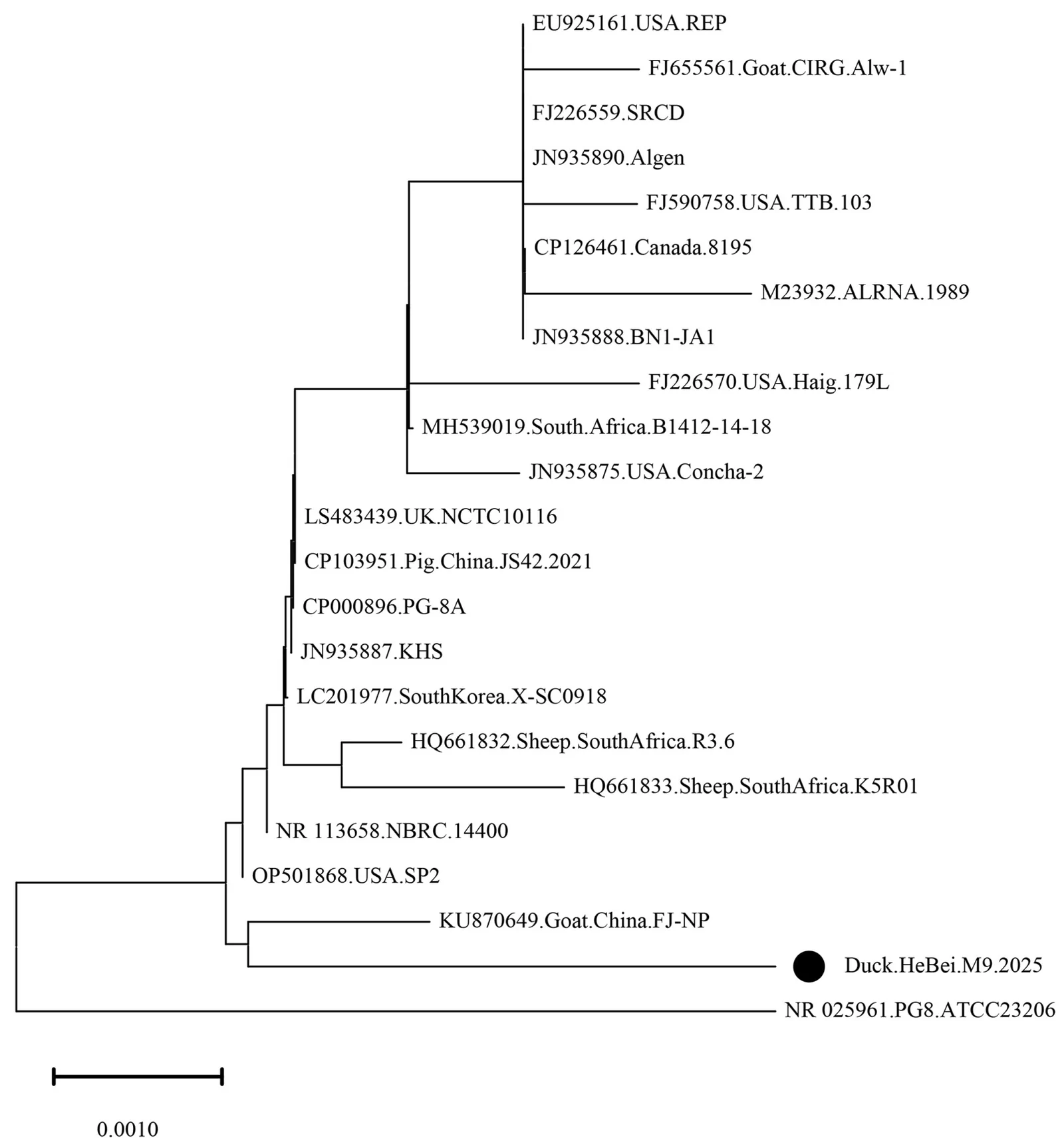
Phylogenetic analysis based on the sequence of 16S rRNA. The black point indicates the isolated strain in this study.

Twenty-two sequences published in NCBI were used to analysis the phylogenetic relationship of the sequence of new isolated strains. Based on the phylogenetic tree, the newly isolated strain (Duck.Hebei.M9.2025) formed a distinct cluster with several reference sequences. Notably, this novel isolate shared a relatively close evolutionary relationship with strains such as CP103951. Pig. China. (No. JS42.2021) and PG-8A (No. CP000896), as evidenced by a short branch length, indicating high genetic similarity. In contrast, it exhibited greater divergence from other reference strains (Figure 3).

### 3.3. MIC Determinations

Antimicrobial susceptibility testing of the *A. laidlawii* strain isolated from ducks was performed against a panel of commonly used antimicrobial agents. The isolate exhibited relatively low MICs for valnemulin (0.0039 μg/mL), tylvalosin (0.0625 μg/mL), tylosin (0.25 μg/mL), and tilmicosin (0.5 μg/mL), whereas relatively high MICs were observed for tiamulin (1 μg/mL), doxycycline (1 μg/mL), chlortetracycline (4 μg/mL), and enrofloxacin (8 μg/mL) (Table 2).

### 3.4. A. laidlawii Strain Could Colonize Ducks with Low Pathogenicity 

During the experimental period, none of the ducks exhibited clinical signs. Postmortem examination revealed no lesions in the lungs or air sacs. *A. laidlawii* was detected in oral and cloacal swabs by PCR. The pathogen was detectable in both types of swabs from 5 to 21 days post-inoculation (dpi). In oral swabs, positive samples numbered 3 at 5 and 7 dpi, 5 at 9 and 14 dpi, 4 at 11 dpi, and 2 at 21 dpi. In cloacal swabs, 2 ducks tested positive at 5 dpi, 3 at 7 dpi, and 4 at 9, 11, and 14 dpi; at 21 dpi, the pathogen remained detectable in 3 ducks. In contrast, no ducks in the control group tested positive at any time point (Table 3).

Bacterial presence was assessed in tissues of the trachea, lung, liver, spleen, heart, and brain. The results showed that the trachea and lung were positive in all infected ducks, whereas the liver and spleen tested positive in only two and one duck(s), respectively. No bacteria were detected in the heart or brain of infected ducks. All tissue samples from the control group remained negative (Table 4).

**Figure 4:**
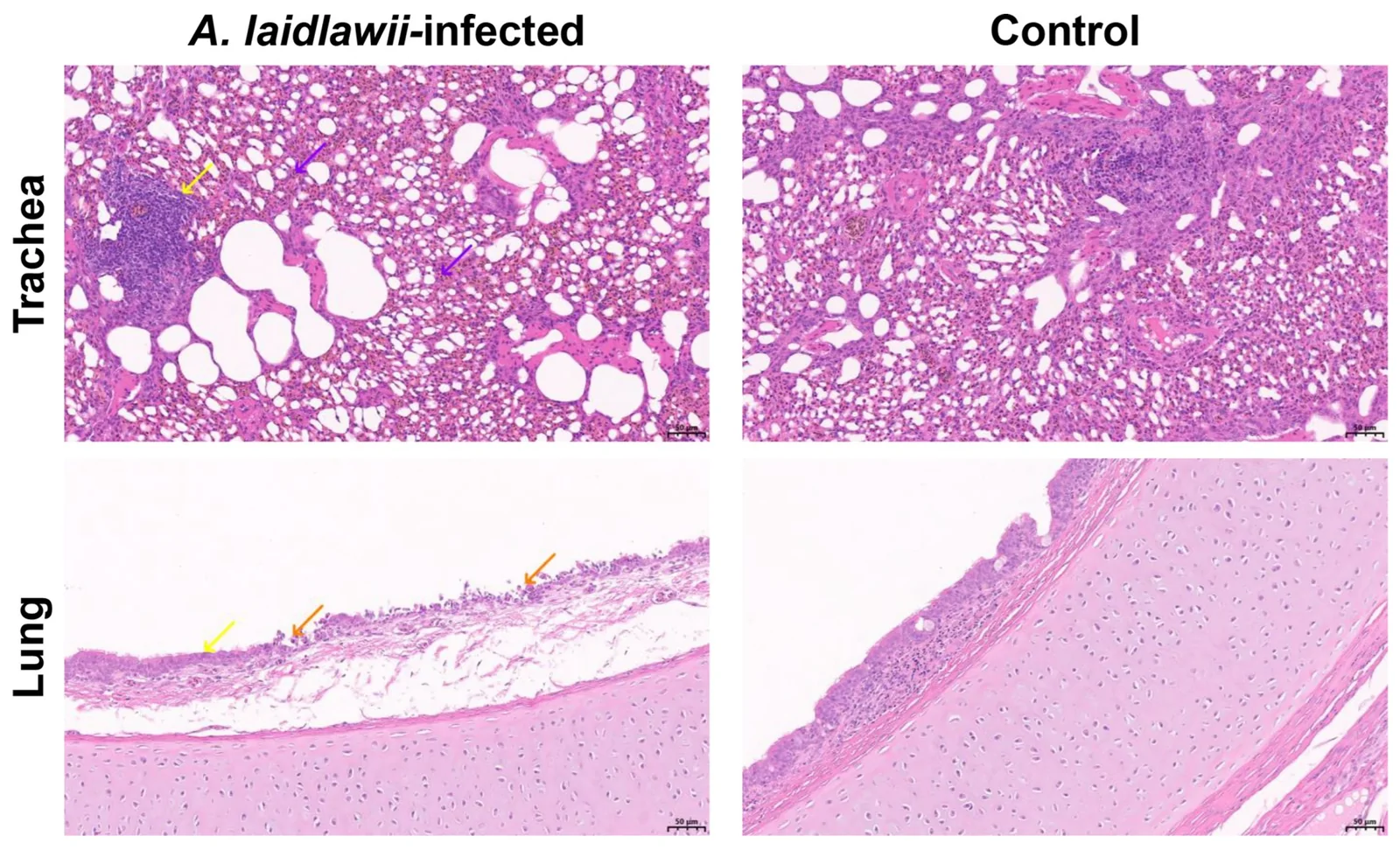
Histopathological analysis of trachea and lung. Trachea in infected group exhibited bronchial and capillary congestion (yellow arrow), and focal lymphocytic infiltration surrounding the bronchi and parabronchi (purple arrows). Lung in infected group exhibited loss of mucosal cilia (yellow arrow) and a small amount of epithelial desquamation (orange arrows). Bar, 50 μm.

Tracheas and lungs were collected for histopathological examination. In the *A. laidlawii*-infected group, the trachea exhibited bronchial and capillary congestion, accompanied by focal lymphocytic infiltration surrounding the bronchi and parabronchi. In the control group, the tracheal mucosa displayed a normal structure, and the number of goblet cells interspersed among the columnar epithelial cells was within the normal range. Histopathological examination of the lungs from infected ducks revealed loss of mucosal cilia, a small amount of epithelial desquamation, exposure of the lamina propria, and a reduced number of goblet cells. In contrast, the lungs of control ducks exhibited a normal alveolar structure without any inflammatory cell infiltration (Figure 4).

## 4. Discussion

As a distinct species within the genus *Mycoplasma*, *A. laidlawii* had been primarily recognized as a causative agent of phytomycoplasmoses and a common contaminant of cell cultures and vaccines [21,22]. Early studies demonstrated that *A. laidlawii* could infect various mammalian species, including goats, lynxes, and pigs, causing respiratory diseases [23,24,25]. In poultry, *A. laidlawii* strains were once isolated from goslings exhibiting severe fibrinous airsacculitis and peritonitis [19]. However, few reports have documented *A. laidlawii* infection in ducks. In the present study, an *A. laidlawii* strain was isolated from ducks exhibiting air sac lesions. Subsequent animal experiments confirmed that the mycoplasma was capable of establishing colonization in the host, accompanied by mild histopathological changes, thereby suggesting that this pathogen has acquired the capacity to infect ducks.

The antimicrobial susceptibility of the new isolate was evaluated against a panel of commonly used antimicrobial agents. The isolate was susceptible to tylvalosin, tylosin, and valnemulin, with relatively low MICs (Table 2), indicating a favorable susceptibility profile. Among the pleuromutilins, valnemulin exhibited markedly lower MICs than tiamulin, a pattern consistent with previous observations in mycoplasmas from other host species [26]. These results suggest that the isolate remains susceptible to the major antimicrobial classes currently used for mycoplasma control in poultry. However, the clinical efficacy of these agents also depends on pharmacokinetic properties, tissue distribution, and the immune status of the host, which should be considered when designing treatment regimens. The antimicrobial susceptibility results provided a basis for guiding clinical antimicrobial therapy.

In contrast to the natural respiratory exposure route, the subcutaneous route with the maximum dosage was selected in the animal experiments. However, none of the infected ducks exhibited clinical signs, and postmortem examination revealed no lesions in the lungs or air sacs. Only mild pathological changes in the trachea and lungs were observed upon histopathological examination (Figure 4), which differed from those seen in clinical cases on the farms. These results indicated that *A. laidlawii* was capable of colonizing ducks, albeit with low pathogenicity. Considering that respiratory clinical signs were observed in the diseased ducks, the subcutaneous inoculation route might not be the most appropriate. Given that only the subcutaneous route was employed in this study, additional inoculation routes should be explored in future studies to attempt to induce detectable clinical signs.

Based on the results above, we hypothesized that *A. laidlawii* infection might act as a predisposing factor for the disease, potentially influenced by stress responses, nutritional factors, environmental conditions, and particularly co-infecting pathogens. From the pneumonic lungs of a lynx (*Lynx canadensis*), researchers isolated both *A. laidlawii* and *Pasteurella multocida*, suggesting that *A. laidlawii* might be considered a possible infectious agent in certain cases of pneumonia [23]. Similarly, in goslings, three mycoplasma species—*A. laidlawii*, *A. axanthum*, and *Mycoplasma gallinarum*—were isolated from geese; however, single infection with *A. laidlawii* alone did not induce clinical signs in animal reversion tests [19]. The pathogenicity of *A. laidlawii* under specific conditions using appropriate animal models warrants further investigation.

Although no similar symptoms were observed in the animal experiments, the reversion tests nevertheless confirmed that the respiratory tract is the more susceptible organ system for *A. laidlawii* in ducks, as all tracheal and lung samples from the infected groups tested positive, whereas fewer samples were positive in other organs, even following subcutaneous inoculation (Table 4). This outcome aligned with the clinical presentation, in which lesions were predominantly localized to the air sacs. Based on these results, we hypothesized that *A. laidlawii* transmission in the farm environment more likely occurs via airborne routes, including aerosols and dust particles, which would be verified in following studies. Furthermore, the pathogen was detectable in most oral and cloacal swabs following infection (Table 3), suggesting that infected ducks might shed the pathogen into the environment, which could potentially facilitate its transmission to other hosts.

## 5. Conclusions

*A. laidlawii* strains were isolated from diseased ducks presenting with air sac lesions. Animal reversion experiments demonstrated that *A. laidlawii* was able to colonize the lungs and trachea of ducks; however, ducks infected solely with this strain did not develop clinical signs comparable to those observed in field cases. These findings indicated that *A. laidlawii* could colonize ducks, and further surveillance is warranted to monitor the variability of pathogens in ducks.

## Figures and Tables

**Table 1:** Primers used in this study.

Name	Sequence (5′—3′)	Product (bp)
AIV F	AAGGGCTTTCACCGAAGAGG	190
AIV R	CCCATTCTCATTACTGCTTC	
aMPV F	TCATGGAGGTCAAGGTAGAG	580
aMPV R	CTCTACCTTGACCCTCCATG	
DAdV F	ATCGAAGTGTCAGATGAAGGAGTG	115
DAdV R	CACCATC-GGGGTTTAGTCGT	
DPV F	CAAACGGGGAGGGCAAAATAAGA	600
DPV R	GTGGTCGCAGGTCCGTAGAGC	
IBV F	CCTGGAAACGAACGGTAGACC	142
IBV R	TAGTGGGCGTCCTA GTGCTG	
DuCV F	GGAGTCGCTGGGAGGAAGAGCC	445
DuCV R	CAAATCATCATACGGCAACC	
RA F	GGATCATTTTGCTTTATCAACAGG	1112
RA R	AGACTTATCTACTGGAGTAGAAACATAATCC	
*Mollicutes* F	GGGAGCAAACACGAT AGATACCCT	270
*Mollicutes* R	TGCACCATC TGTCACTCTGTTAACCTC	
16S rRNA F	AGAGTTGATCMTGGCTCAG	1464
16S rRNA R	TACGGYTACCTTGTTACGACTT	
*A. laidlawii* F	TAATCCTGTTTGCTCCCCAC	505
*A. laidlawii* R	AGCCGGACTGAGAGGTCTAC	

**Table 2:** MIC of *A. laidlawii* strain isolated from ducks.

Drugs	MIC Value (μg/mL)
Tylvalosin	0.0625
Tylosin	0.25
Tiamulin	1
Valnemulin	0.0039
Doxycycline	1
Chlortetracycline	4
Enrofloxacin	8
Tilmicosin	0.5

**Table 3:** Detection of *A. laidlawii* in oral and cloacal swabs after infection.

Group	Swabs	Positive Samples at Each Timepoint (dpi)							
		1	3	5	7	9	11	14	21
*A. laidlawii*-infected	oral	0/10	0/10	3/10	3/10	5/10	4/10	5/10	2/10
	cloacal	0/10	0/10	2/10	3/10	4/10	4/10	4/10	3/10
Control	oral	0/10	0/10	0/10	0/10	0/10	0/10	0/10	0/10
	cloacal	0/10	0/10	0/10	0/10	0/10	0/10	0/10	0/10

**Table 4:** Detection of *A. laidlawii* in tissues at 21 dpi.

Group	Positive Tissues					
	Trachea	Lung	Liver	Spleen	Heart	Brain
*A. laidlawii*-infected	10/10	10/10	2/10	1/10	0/10	0/10
Control	0/10	0/10	0/10	0/10	0/10	0/10

## Data Availability

The original contributions presented in this study are included in the article. Further inquiries can be directed to the corresponding author.

## Supplementary Materials

The following supporting information can be downloaded at: https://www.mdpi.com/article/10.3390/vetsci13101056/s1. Table S1: Sample information of diseased ducks from Heibei province.

## Abbreviations

The following abbreviations are used in this manuscript:

SPF	specific-pathogen-free
AIV	avian influenza virus
aMPV	avian metapneumovirus
DAdV	Duck Adenovirus
DPV	Duck Plague Virus
IBV	Infectious Bronchitis Virus
DuCV	Duck Circovirus
RA	Riemerella anatipestifer
dpi	days post-inoculation
