# Supplementary material for: Emergence of Acholeplasma laidlawii Infection in Ducks: Isolation and Colonization Potential
Source: Vet Sci. 2026 Oct 8;13(10):1056. doi: 10.3390/vetsci13101056 (PMC13709394; doi:10.3390/vetsci13101056)
Supplement: Supplementary file 1 [file vetsci-13-01056-s001.zip › vetsci-4570139-supplementary.pdf]

**TABLE S1 |** Sample information of diseased ducks from Heibei province

| No. | Farmer | District | Age of ducks (days) | Flock size | Morbidity (%) | Sample quantity | Mycoplasma isolation |
|---|---|---|---|---|---|---|---|
| 1 | LIU | Cangzhou | 23 | 5000 | 20 | 5 | Positive |
| 2 | QIU | Hengshui | 15 | 3000 | 20 | 6 | Positive |
| 3 | WANG | Cangzhou | 11 | 5000 | 30 | 6 | Positive |
| 4 | LI | Baoding | 32 | 2000 | 20 | 4 | Positive |
| 5 | CANG | Xingtai | 25 | 5000 | 10 | 5 | Positive |
| 6 | LIN | Hengshui | 5 | 7000 | 30 | 10 | Positive |
| 7 | MA | Baoding | 31 | 3000 | 20 | 4 | Positive |
| 8 | ZHAO | Cangzhou | 28 | 3000 | 10 | 4 | Positive |

Ducks in all farms were commercial meat ducks. All ducks were euthanized prior to postmortem examination, which was conducted in the laboratory. Tissue samples (livers, lungs, airsacs, and tracheas) were collected for subsequent bacterial identification and isolation. The morbidity rate was calculated as the proportion of ducks exhibiting clinical signs or lesions, based on anatomical examination.
